# Myocardial protection against global ischemia with Krebs-Henseleit buffer-based cardioplegic solution

**DOI:** 10.1186/1749-8090-8-60

**Published:** 2013-04-02

**Authors:** Sarkis M Minasian, Michael M Galagudza, Yuri V Dmitriev, Dmitry I Kurapeev, Timur D Vlasov

**Affiliations:** 1 Institute of Experimental Medicine, V. A. Almazov Federal Heart, Blood and Endocrinology Centre, Saint Petersburg, Russia; 2 Department of Pathophysiology, I. P. Pavlov Federal Medical University, Saint Petersburg, Russia

**Keywords:** Heart, Ischemia/reperfusion injury, Myocardial protection/cardioplegia

## Abstract

**Background:**

The Krebs-Henseleit buffer is the best perfusion solution for isolated mammalian hearts. We hypothesized that a Krebs-Henseleit buffer-based cardioplegic solution might provide better myocardial protection than well-known crystalloid cardioplegic solutions because of its optimal electrolyte and glucose levels, presence of buffer systems, and mild hyperosmolarity.

**Methods:**

Isolated Langendorff-perfused rat hearts were subjected to either global ischemia without cardioplegia (controls) or cardioplegic arrest for either 60 or 180 min, followed by 120 min of reperfusion. The modified Krebs-Henseleit buffer-based cardioplegic solution (mKHB) and St. Thomas’ Hospital solution No. 2 (STH2) were studied. During global ischemia, the temperatures of the heart and the cardioplegic solutions were maintained at either 37°C (60 min of ischemia) or 22°C (moderate hypothermia, 180 min of ischemia). Hemodynamic parameters were registered throughout the experiments. The infarct size was determined through histochemical examination.

**Results:**

Cardioplegia with the mKHB solution at moderate hypothermia resulted in a minimal infarct size (5 ± 3%) compared to that in the controls and STH2 solution (35 ± 7% and 19 ± 9%, respectively; *P* < 0.001, for both groups vs. the mKHB group). In contrast to the control and STH2-treated hearts, no ischemic contracture was registered in the mKHB group during the 180-min global ischemia. At normothermia, the infarct sizes were 4 ± 3%, 72 ± 6%, and 70 ± 12% in the mKHB, controls, and STH2 groups, respectively (*P* < 0.0001). In addition, cardioplegia with mKHB at normothermia prevented ischemic contracture and improved the postischemic functional recovery of the left ventricle (*P* < 0.001, vs. STH2).

**Conclusions:**

The data suggest that the Krebs-Henseleit buffer-based cardioplegic might be superior to the standard crystalloid solution (STH2).

## Background

Myocardial protection against global ischemia-reperfusion injury during open-heart surgery with cardiopulmonary bypass and cardioplegic arrest remains a challenging problem [1]. Despite recent advances, such factors as progressive aging of patients, the occurrence of comorbidities, and preexisting severe myocardial dysfunction require further improvements in intraoperative cardiac protection [2,3].

Currently, it is well established that isothermic blood cardioplegia provides robust myocardial protection and results in lesser myocardial injury, as detected on biochemical examination, and better outcomes than cold crystalloid cardioplegia [4-6]. However, blood cardioplegia has some limitations, including increased risk of systemic hyperkalemia [7] and technical problems in pediatric, particularly neonatal, cardiac surgery [8,9]. Surgical treatment of complex congenital heart defects in neonates is usually associated with an extended duration of anoxia, therefore requiring particularly careful myocardial protection. Consequently, the development of novel and improved crystalloid cardioplegic solutions may be necessary. Given that the Krebs-Henseleit buffer (KHB) is considered the best option for perfusion of the isolated mammalian heart, KHB-based cardioplegia may be an interesting option. In the literature, only 1 report is available on the feasibility of myocardial protection by a modified KHB [10]. In this study, a modified KHB with increased potassium (20 mmol/L) and calcium concentrations (0.1–2.5 mmol/L) was used for continuous coronary perfusion of the isolated rat heart for 180 min at 37°C. Minimal myocardial injury was found when the hearts were perfused with a solution containing 1.5 mmol/L calcium.

We hypothesized that a KHB-based crystalloid cardioplegic solution provides superior myocardial protection compared to that provided by a well-accepted solution, that is, St Thomas’ Hospital cardioplegic solution No. 2 (STH2). The purpose of the present study was to compare the protective effect of KHB-based cardioplegia with that of STH2-based cardioplegia during normothermic (37°C) and moderate hypothermic (22°C) cardiac arrest.

## Methods

### Animals

Adult male Wistar rats (body weight, 250–320 g) were used throughout the experiments. All the animals received humane care in accordance with the European Convention on Animal Care regulations. The study was approved by the ethics committee of V. A. Almazov Federal Heart, Blood and Endocrinology Centre, Saint Petersburg, Russian Federation.

### Perfusion of isolated hearts

The rats were anesthetized with sodium pentobarbital (60 mg/kg intraperitoneally). Heparin was not administered before heart excision. Each heart was excised via bilateral thoracotomy and perfused through the ascending aorta with KHB consisting of the following (in mmol/L), at a constant pressure of 85 mm Hg: NaCl, 118.5; KCl, 4.7; NaHCO_3_, 25; KH_2_PO, 1.2; MgSO_4_, 1.2; glucose, 11; and CaCl_2_, 1.5. Perfusion pressure was maintained by gravity, that is, by using a water-jacketed double-walled glass column connected to the aortic cannula via a 3-way stopcock. The oxygenation of KHB was performed with carbogen (95% O_2_ plus 5% CO_2_) delivered through the inverted fritted glass filter to maintain pO_2_ ≥ 500 mm Hg. The time interval between the opening of the thoracic cavity and initiation of perfusate flow to the heart was < 80 s.

Heart function was stabilized for 15 min before cardioplegic arrest. Left ventricular systolic (LVSP) and end-diastolic pressures (LVEDP) were measured isovolumetrically using a nonelastic polyethylene balloon introduced into the left ventricle via the left atrium. The balloon was coupled to an insulin syringe and inflated with 0.4–0.6 mL of boiled water to obtain a LVEDP < 10 mm Hg during stabilization. Software (PhysExp Gold, Cardioprotect Ltd., Saint Petersburg, Russian Federation) was used to process the pressure wave recorded using a miniature pressure transducer (Baxter International, Deerfield, Ill., USA) and produce every minute a value corresponding to the mean LVEDP and LVSP. Left ventricular developed pressure (LVDP) was calculated as LVSP − LVEDP. Heart rate (HR) was derived from the pressure wave. Coronary flow rate (CFR) was measured by timed collection of the perfusate outflow.

The temperatures of the heart and cardioplegic solutions during global ischemia were maintained at either 37°C (normothermia) or 22°C (moderate hypothermia). The heart temperature was maintained between cardioplegia infusions by means of heart immersion into a buffer-filled water-jacketed glass chamber. The temperature in the chamber was maintained using a thermocirculator. The myocardial temperature was monitored using a miniature temperature probe inserted into the right ventricular cavity through a small incision in the pulmonary artery.

In the normothermia series, the cardioplegic solution was administered antegradely at a constant pressure of 85 mm Hg at the start and after 20 and 40 min of global ischemia for 4, 2, and 2 min, respectively. The amount of cardioplegic solution given during each administration was approximately 30–40% more than the baseline CFR, possibly owing to the rapid cessation of cardiac contractions.

In the hypothermia series, 9 infusions of the cardioplegic solutions were performed during each 20 minutes of global ischemia. The initial episode lasted for 60 s, and every subsequent episode lasted for 30 s. The duration of cardioplegia delivery was selected on the basis of previous work indicating that optimal myocardial protection with STH2 in the rat heart can be achieved when the solution is infused for duration ≥ 30 s [11]. In the hypothermia series, the cardioplegia dosage per episode was lower than that in the normothermia series. Along with an extended duration of global ischemia, the cardioplegia dosage was intentionally lowered to obtain a greater amount of myocardial necrosis in the hypothermic controls, from which we could derive stronger conclusions about the extent of the protective effect of each of the various cardioplegic solutions tested.

### The cardioplegic solutions

The chemical composition of the tested cardioplegic solutions is presented in Table 1. The chemical composition of the KHB was modified according to the following to create the cardioplegic solution: [K^+^] was raised from 5.9 to 25 mmol/L; [Mg^2+^] was raised from 1.2 to 16 mmol/L; and [Ca^2+^] was reduced from 1.5 to 0.3 mmol/L. The cardioplegic solutions were not gassed with oxygen.

**Table 1:** Chemical composition of the tested crystalloid cardioplegic solutions

**Component, mmol/l**	**Krebs-Henseleit buffer-based cardioplegic solution**	**St Thomas’ Hospital cardioplegic solution №2**
Sodium	143	120
Potassium	25	16
Calcium	0.3	1.2
Magnesium	16	16
Glucose	11	–
Bicarbonate	25	10
Dihydro orthophosphate	1.2	–
рН	7.8	7.8
Osmolarity, mOsm/l	380	310

### Experimental protocol and exclusion criteria

In the hypothermia (22°C) series, hearts were allocated into 1 of the following 3 groups:

1. Controls (CON-22, n = 8): global ischemia at 22°C (no cardioplegia) for 180 min followed by 120 min of reperfusion at 37°C.

2. Cardioplegia with modified KHB solution (mKHB-22, n = 7): cardiac arrest for 180 min followed by 120 min of reperfusion at 37°C.

3. Cardioplegia with STH2 (STH2-22, n = 8): cardiac arrest for 180 min followed by 120 min of reperfusion at 37°C.

In the normothermia (37°C) series, hearts were allocated to 1 of the following 3 groups:

1. Controls (CON-37, n = 8): global ischemia (no cardioplegia) for 60 min followed by 120 min of reperfusion.

2. Cardioplegia with modified KHB solution (mKHB-37, n = 5): cardiac arrest for 60 min followed by 120 min of reperfusion.

3. Cardioplegia with STH2 (STH2-37, n = 6): cardiac arrest for 60 min followed by 120 min of reperfusion.

LVSP, LVEDP, HR, and CFR were measured 5 min before global ischemia and at the 5th, 30th, 60th, and 120th min of reperfusion. In addition, the left ventricular (LV) pressures were measured at the 5th, 15th, 25th, 35th, 45th, and 55th min of global ischemia in the normothermia series and during each 20 min of global ischemia in the hypothermia series.

Any heart with a HR < 220 beats/min and a CFR > 18 or < 8 mL/min by the end of stabilization was excluded from the study. Hearts failing to show a LVDP > 100 mm Hg when the LVEDP was maintained at < 10 mm Hg were also excluded.

### Infarct size determination

At the end of reperfusion, the hearts were rapidly cut into 4 equally spaced transverse slices. The slices were immersed in 1% solution of 2,3,5-triphenyltetrazolium chloride (TTC) for 15 min at 37°С. The stained slices were photographed with a digital camera for further determination of the TTC-negative (infarcted) area. After computer planimetry (Photoshop CS), the infarct size was expressed as a percentage of total ventricular area minus the cavities, and the mean value of all the sections in a heart was used for the statistical analysis.

### Statistical analysis

The continuous functional data and infarct size in the text are expressed as mean ± standard deviation (SD). The sample size per group was determined using the following parameters: SD values calculated on the basis of previous studies, desired confidence level (95%), and acceptable difference in outcome between the groups (Statistics Calculator). The statistical analysis was performed using the SPSS 20.0 software package. The Kruskal-Wallis test was used to determine differences in infarct size, followed by pairwise intergroup comparisons performed using nonparametric Mann–Whitney *U* test. Differences in continuous data were tested using repeated-measures analysis of variance, followed by a Tukey post hoc test. *P* ≤ 0.05 was considered significant.

## Results

### Exclusions and rates of persistent ventricular fibrillation/asystole during reperfusion

Of 42 hearts perfused, 4 were excluded during stabilization (1 in the CON-22 group, 2 in the STH2-22 group, and 1 in the CON-37 group). The frequencies of persistent ventricular fibrillation or asystole during reperfusion in the hypothermia series were 0/7, 0/7, and 0/6 in the CON-22, mKHB-22, and STH2-22 groups, respectively. In the normothermia series, the frequencies were 3/7, 0/5, and 0/6 in the CON-37, mKHB-37, and STH2-37 groups, respectively. The hearts with persistent ventricular fibrillation/asystole during reperfusion were not included in the analysis of postischemic LV function but were used for the determination of infarct size.

### Ischemic contracture

**Figure 1:**
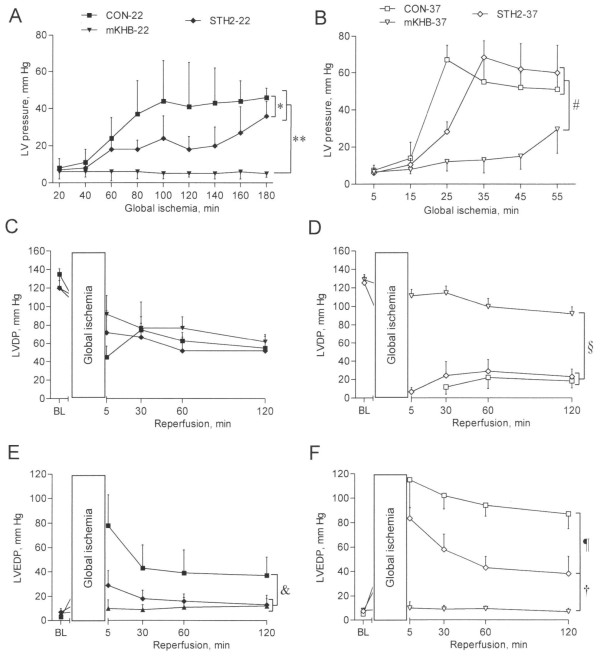
**Hemodynamic parameters in the isolated rat hearts subjected to global ischemia under either a hypothermic or normothermic condition (22°C or 37°C, respectively) followed by 120 min of normothermic reperfusion.** Ischemic contracture in the hypothermia (**A**) and normothermia series (**B**); left ventricular developed pressure (LVDP) in the hypothermia (**C**) and normothermia series (**D**); and left ventricular end-diastolic pressure (LVEDP) in the hypothermia (**E**) and normothermia series (**F**). Group legends in the hypothermia series: the controls subjected to 180-min ischemia and 120-min reperfusion (CON-22), cardioplegia with the modified Krebs-Henseleit buffer-based cardioplegic solution (mKHB-22), and cardioplegia with St. Thomas’ Hospital cardioplegic solution No. 2 (STH2-22). Group legends in the normothermia series: controls subjected to 60-min ischemia and 120-min reperfusion (CON-37), cardioplegia with the modified Krebs-Henseleit buffer-based cardioplegic solution (mKHB-37), and cardioplegia with St. Thomas’ Hospital cardioplegic solution No. 2 (STH2-37). BL, baseline values. The data are the mean ± SD values. *P < 0.01; **P < 0.001; ^&^*P* < 0.05; ^#^*P* < 0.001; ^§^*P* < 0.0001; ^¶^*P* < 0.0001; ^†^*P* < 0.001.

The dynamics of LV pressure during global ischemia and cardioplegia in the hypothermia and normothermia series are shown in Figure 1A and B, respectively. Ischemic contracture was defined as at least a 3-fold increase in LV pressure at any time point of the ischemic period relative to the LV pressure after 5 min of z`ischemia. No ischemic contracture was found in the mKHB-22 group. The LV pressures during ischemia were significantly higher in the other hypothermic groups than in the mKHB-22 group (*P* < 0.001, Figure 1A). In the normothermia series, the strongest ischemic contracture was found in the CON-37 and STH2-37 groups (Figure 1B). Ischemic contracture was significantly lower in the mKHB-37 group than in the other normothermic groups (e.g., *P* = 0.0004, vs. the STH2-37 group).

### Left ventricular developed pressure and end-diastolic pressure

**Figure 2:**
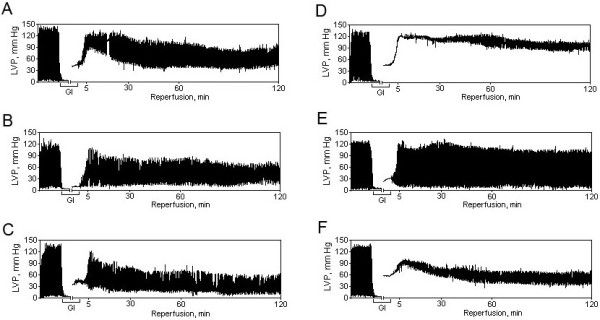
**Representative LV pressure recordings demonstrating different patterns of LV function recovery in CON-22 (A), mKHB-22 (B), STH2-22 (C), CON-37 (D), mKHB-37 (E), and STH2-37 (F) groups.** See Figure 1 for the group legends. GI, global ischemia; LVP, left ventricular pressure.

The baseline LVDPs and LVEDPs were similar between all the groups. No spontaneous mechanical activity was registered during ischemia, including the cardioplegia infusion episodes, except at the beginning of the first cardioplegia episode. In the control groups (at 22°C and 37°C), asystole started soon after the onset of global ischemia, though a bit later than after the start of cardioplegia (2–3 min vs. 10–20 s). Representative LV pressure recordings demonstrating different patterns of postischemic LV function recovery are presented in Figure 2. In the hypothermia groups, the LVDP recovery was not different between the CON-22, mKHB-22, and STH2-22 groups (Figure 1C). Among the normothermia groups, the CON-37 group showed no cardiac contraction during the initial 5 min of reperfusion. The recovery of LVDP in the STH2-37 group was not different from that in the CON-37 group (Figure 1D). However, LVDP recovery was significantly greater in the mKHB-37 group than in the CON-37 and STH2-37 groups (*P* < 0.0001).

In the hypothermia series, the postischemic LVEDP was significantly lower in the mKHB-22 and STH2-22 groups than in the CON-22 group (*P* < 0.05, Figure 1E). In the normothermia series, the postischemic LVEDP was significantly lower in the STH2-37 group than in the CON-37 group (*P* < 0.0001, Figure 1F). The postischemic LVEDP remained normal only in the mKHB-37 group (*P* < 0.001, vs. the STH2-37 group; Figure 1F).

### Coronary flow rate and heart rate

The preischemic values of CFR and HR did not differ between the groups (Tables 2 and 3). No important intergroup and intragroup differences in CFR or HR were found.

**Table 2:** The values of coronary flow rate (ml/min) in the experimental groups

**Groups**	**Baseline**	**Reperfusion, min**			
		5	30	60	120
CON-22 (n=7)	11.9±2.3	3.8±1.5	5.4±1.6	6.5±1.9	5.8±2.6
mKHB-22 (n=7)	10.2±2.6	4.5±2.3	6.7±1.6	6.1±1.8	4.2±1.7
STH2-22 (n=6)	13.2±3.5	5.3±2.5	6.7±2.2	7.5±4.6	5.5±2.1
CON-37 (n=7)	12.4±3.2	4.1±1.3	4.3±1.9	4.1±1.7	3.8±1.2
mKHB-37 (n=5)	10.4±1.9	6.6±4.0	6.2±1.7	6.3±0.6	5.2±1.4
STH2-37 (n=6)	11.2±1.8	5.6±3.9	4.6±2.1	3.3±0.7	3.1±0.9

Data are mean ± SD. Group legends in hypothermia series: controls subjected to 180-min ischemia and 120-min reperfusion (CON-22), cardioplegia with modified Krebs-Henseleit buffer-based cardioplegic solution (mKHB-22), cardioplegia with St Thomas’ Hospital cardioplegic solution №2 (STH2-22). Group legends in normothermia series: controls subjected to 60-min ischemia and 120-min reperfusion (CON-37), cardioplegia with modified Krebs-Henseleit buffer-based cardioplegic solution (mKHB-37), cardioplegia with St Thomas’ Hospital cardioplegic solution №2 (STH2-37).

**Table 3:** The values of heart rate (beats/min) in the experimental groups

**Groups**	**Baseline**	**Reperfusion, min**			
		5	30	60	120
CON-22 (n=7)	243±16	215±21	218±22	238±28	222±35
mKHB-22 (n=7)	253±25	218±32	219±29	246±34	251±28
STH2-22 (n=6)	256±14	227±28	216±22	245±35	236±32
CON-37 (n=7)	250±23	-	187±39	212±52	242±68
mKHB-37 (n=5)	268±21	191±39	195±24	211±32	232±58
STH2-37 (n=6)	269±13	180±35	187±37	223±47	224±12

Data are mean ± SD. Group legends in hypothermia series: controls subjected to 180-min ischemia and 120-min reperfusion (CON-22), cardioplegia with modified Krebs-Henseleit buffer-based cardioplegic solution (mKHB-22), cardioplegia with St Thomas’ Hospital cardioplegic solution №2 (STH2-22). Group legends in normothermia series: controls subjected to 60-min ischemia and 120-min reperfusion (CON-37), cardioplegia with modified Krebs-Henseleit buffer-based cardioplegic solution (mKHB-37), cardioplegia with St Thomas’ Hospital cardioplegic solution №2 (STH2-37).

“-” - persistent ventricular fibrillation/asystole in all hearts.

### Myocardial infarct size

**Figure 3:**
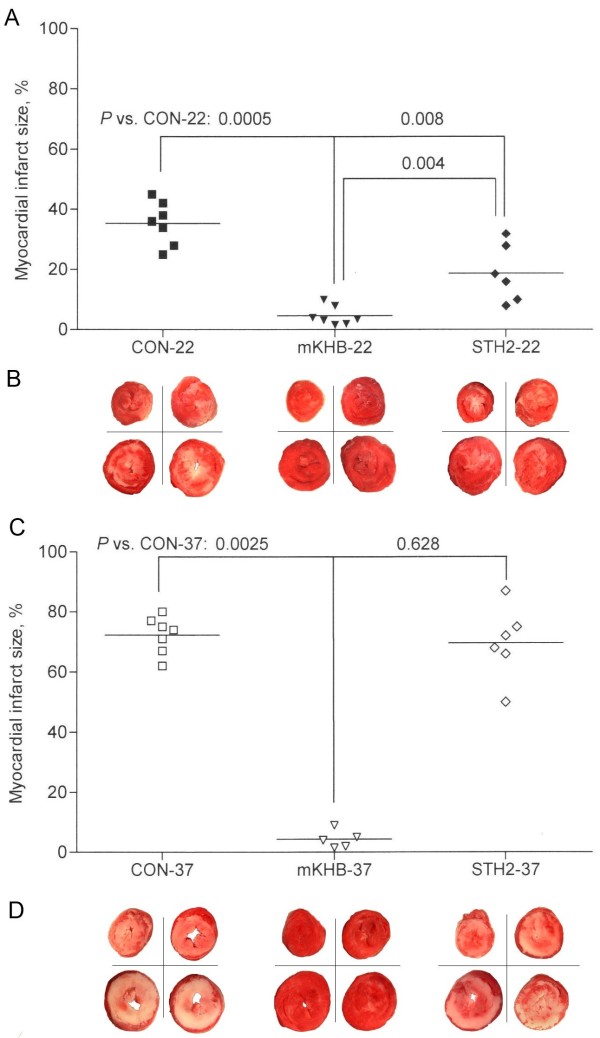
**Myocardial infarct size after the 120-min reperfusion of the Langendorff-perfused rat hearts that were subjected to 60 or 180 min of global ischemia, sectioned, and stained with triphenyltetrazolium chloride.** Infarct size in the hypothermia (**A**) and normothermia series (**C**). The data are presented as dot plots with median values. The triphenyltetrazolium chloride-stained heart slices selected from the representative experiments in each group in the hypothermia (**B**) and normothermia series (**D**). See Figure 1 for the group legends.

The myocardial infarct size is depicted in Figure 3. Control ischemia with moderate hypothermia for 180 min without cardioplegia (CON-22) resulted in an infarct size of 35 ± 7% after reperfusion for 120 min (Figure 3A,B). In the hypothermia series, cardioplegia with mKHB and STH2 reduced the infarct size in comparison with the controls (5 ± 3% and 19 ± 9%, respectively; *P* = 0.0005 and 0.008, vs. the CON-22 group, respectively). In addition, the infarct size was significantly smaller in the mKHB group than in the STH2 group (*P* = 0.004). In the normothermia series, the CON-37 and STH2-37 groups had no difference in infarct size (72 ± 6% and 70 ± 12%, respectively; Figure 3C,D). However, mKHB cardioplegia reduced the infarct size (4 ± 3%; *P* = 0.0025, vs. the CON-37 and STH2-37 groups, Figure 3C,D).

## Discussion

The present study suggested that an mKHB-based cardioplegic solution had stronger infarct-limiting effect and preserved postischemic ventricular function better than STH2 after normothermic and moderately hypothermic cardioplegia.

Myocardial temperature is a key factor in cardiac protection against ischemia [12,13]. Hypothermia slows down all metabolic processes, contributing to prolonged tissue survival during ischemia [12,14]. However, deep hypothermia itself may exert certain injurious effects [15]. During the recent decades, the strategy of intraoperative myocardial protection has changed from deep hypothermic toward normothermic blood cardioplegia. In particular, a number of clinical trials demonstrated excellent results with the use of normothermic blood cardioplegia versus cold crystalloid cardioplegia [4-6]. In normothermic cardioplegia protocols, the temperature of the blood-based cardioplegic solution is maintained at approximately 33°C to 37°C [16]. However, many centers continue to use either cold crystalloid or cold blood cardioplegia in their routine practice [17,18].

Documented experience with warm crystalloid cardioplegia is lacking. Hence, we conducted the present study with the hope of contributing additional information about the efficacy of mKHB-based cardioplegia by elucidating its effects during normothermia and moderate hypothermia. With temperatures of 5–10°C, the isolated perfused rat hearts required cardioplegia arrest for >6 h to obtain a significant ischemic injury. Therefore, we chose the temperature of 22°С. This is beyond the clinical relevance for cardioplegia, but it might be an interesting future study for organ preservation for transplantation. At both temperatures, mKHB-based cardioplegia showed significantly improved cardioprotection compared to that with STH2-based cardioplegia. This finding may be attributed to a number of factors.

Factor 1. A positive effect might be ascribed to the presence of glucose at a concentration of 11 mmol/L. Previous experiments on isolated working rat hearts subjected to hypothermic multidose cardioplegic arrest have demonstrated that the supplementation of STH2 with 11 mmol/L glucose resulted in better postischemic recovery in comparison with lower (1 mmol/L) and higher (20 and 50 mmol/L) glucose concentrations in STH2 [19]. Glucose at a concentration of 11 mmol/L has been demonstrated to stimulate glycolytic ATP production during ischemia, whereas the use of higher glucose concentrations suppressed this process. In addition, excessive glucose levels may lead to cardiac myocyte injury because of osmotic overload and increased risk of intracellular edema.

Factor 2. Although the optimal calcium concentration is yet to be determined, we used 0.3 mmol/L in the mKHB-based cardioplegic solution in this study. The calcium content in the currently available cardioplegic solutions varies from zero to physiological and even increased levels. In a model of normothermic continuous coronary perfusion of the isolated rat heart, Nakamura et al. [10] demonstrated that the optimal calcium concentration in mKHB-based solution is 1.5 mmol/L. It should be noted, however, that the heart subjected to continuous coronary perfusion is much more sensitive to calcium-induced injury at reperfusion as compared with the heart undergoing intermittent cardioplegia, suggesting that a lower calcium concentration (e.g., 0.3 mmol/L) might contribute to the attenuation of myocardial injury via the prevention of intracellular calcium overload. This hypothesis has been further supported by the finding that the decrease in calcium concentration in STH2 to 0.3–0.9 mmol/L resulted in enhanced postischemic recovery of aortic flow in the isolated working rat heart [20]. In this study cardioplegic solutions were infused each 30 minute during global ischemia at 20°C for 300 minutes. The use of acalcemic STH2 has been associated with severe myocardial injury owing to the calcium paradox. Of note, cardiac reperfusion after STH2 cardioplegia and 25-min normothermic arrest with perfusate containing 0.25–1.0 mmol/L of calcium was not associated with additional protection in comparison with cardioplegia itself [21].

Factor 3. The mKHB-based cardioplegic solution contains bicarbonate and phosphate buffers that have equal total inorganic buffer capacity to that of human plasma. Such buffers minimize rapid changes in extracellular and intracellular pH values during the bouts of ischemia-reperfusion.

Factor 4. The mKHB solution developed for the present study had an osmolarity of 380 mosm/L, which is 22.6% higher than that in STH2. This mild increase in osmolarity may prevent myocardial edema during early reperfusion.

The dynamics of LV pressure during global ischemia is commonly used as a sensitive indicator of ischemic myocardial injury [22]. In the present study, the use of mKHB in the hypothermic mode prevented completely ischemic contracture. In addition, normothermic mKHB-based cardioplegia yielded the lowest levels of LV pressure during ischemia among all of the normothermic options applied. Meanwhile, using mKHB-based cardioplegia in normothermic conditions, we observed a significant increase in LV pressure during the final 15 min of global ischemia, which signaled the onset of ischemic contracture. This fact indicates that the cardioprotective potential of the modified mKHB solution may fail by this time.

To the best of our knowledge, the KHB has not previously been considered as a basis for a cardioplegic solution, at least for intermittent antegrade cardioplegia. Some of the studies were aimed at improving myocardial protection with STH2 by means of different modifications. In addition to the aforementioned changes in glucose and calcium levels, improved protection was demonstrated after oxygenation of STH2 [23] as well as after supplementation with high-energy phosphates [24] or antioxidants [25]. A recent study with the Langendorff-perfused rat heart demonstrated that the addition of inward rectifier potassium channel agonist zacopride to the STH2 resulted in significant reduction of infarct size and amelioration of LV function after 45 min of normothermic arrest [26].

During the 2 recent decades, several crystalloid cardioplegic solutions with enhanced cardioprotective properties have been developed and experimentally tested. The protective efficacy of these novel solutions was compared to that of the gold standard of crystalloid cardioplegia, that is, STH2. For example, the extracellular cardioplegic solution MBS containing 10 mmol/L glucose, 10 mmol/L l-aspartic acid, and 5 mmol/L lactobionic acid was shown to be superior to STH2 in the isolated working rat heart subjected to prolonged hypothermic arrest [27]. Another crystalloid cardioplegic solution has been developed on the basis of the non-phosphate-buffered perfusion solution Aqix® RS-I [28]. This magnesium-based solution provided better functional LV recovery than STH2 after either single or multiple administrations in the Langendorff-perfused rat heart with global ischemia at normothermia. The optimal magnesium concentration was found to be 25 mmol/L, whereas the potassium content was kept at 5 mmol/L. In our study, the use of 16 mmol/L magnesium was well justified by the presence of 25 mmol/L potassium in mKHB. The beneficial effect of the Aqix® RS-I-based cardioplegic solution could be explained by the presence of 10 mmol/L glucose and some other substrates/cofactors such as glutamate, aspartate, choline, and cocarboxylase. Thus, we speculate that the protective ability of mKHB could be further enhanced by the addition of these essential components.

The present study has several methodological limitations. First, the many modifications and differences in the composition of the mKHB-based cardioplegia made it different from the STH2 solution. Hence, we could not specify which aspect made the mKHB solution superior to STH2. However, the mKHB solution showed superior cardioprotective abilities, in function and infarct size reduction, in normothermia and moderate hypothermia. Second, the numbers of hearts, especially in the normothermia series, were rather small. Nevertheless, we were able to clearly demonstrate the effects of the various solutions. Third, the cardioplegic solutions were delivered at a relatively high pressure of 85 mm Hg, which might have potentially resulted in endothelial damage. In the clinical setting, cardioplegia is usually delivered to the aorta at pressures varying from 60 to 80 mm Hg. Although the perfusate pressure was slightly higher than the usual, it was equal in both cardioplegic solutions tested. Future studies are required to address these important issues. Finally, the volume of cardioplegic solutions infused was not measured.

The evidence obtained from our results may be considered adequate to prove the superiority of the new mKHB solution to STH2. However, one may argue that the isolated rat heart model is far from the clinical situation, and it is necessary to test the new solution in more relevant models and against blood cardioplegia. In this regard, it is important to note that the present model is the model from which the St. Thomas’ Hospital cardioplegic solution was developed.

## Conclusions

The present data suggested that the KHB-based cardioplegic solution might be superior to the standard crystalloid solution during moderate hypothermia and normothermia.

## Abbreviations

LV: Left ventricle; KHB: Krebs-Henseleit buffer; mKHB: modified Krebs-Henseleit buffer-based cardioplegic solution; STH2: St Thomas’ Hospital cardioplegic solution No. 2; LVEDP: Left ventricular end-diastolic pressure; LVDP: Left ventricular developed pressure; HR: Heart rate; CFR: Coronary flow rate.

## Competing interests

The authors declare that they have no competing interests.

## Authors’ contributions

SMM and MMG performed the experiments, analyzed and interpreted the data, performed the statistical analysis, and wrote the manuscript; YVD, DIK and TDV contributed to the design the study, helped to draft the final manuscript and added important comments to the paper. All authors read and approved the final manuscript.
